# Healthcare professionals’ practice and interactions in older peoples’ cross-sectoral clinical care trajectories when acutely hospitalized - a qualitative observation study

**DOI:** 10.1186/s12913-021-06953-9

**Published:** 2021-09-09

**Authors:** Maiken Hjuler Persson, Christian Backer Mogensen, Jens Søndergaard, Helene Skjøt-Arkil, Pernille Tanggaard Andersen

**Affiliations:** 1 Emergency Department, Hospital Sønderjylland, Kresten Philipsens Vej 15, 6200 Aabenraa, Denmark; 2 Research Unit for Health Promotion, Department of Public Health, University of Southern Denmark, Degnevej 14, 6705 Esbjerg, Denmark; 3 Department of Regional Health Research, Winsløwparken 19,3, 5000 Odense, Denmark; 4 University of Southern Denmark, Odense, Denmark; 5 Research Unit of General Practice, J. B. Winsløws Vej 9A, 5000 Odense, Denmark; 6 Research Unit for Health Promotion, Department of Public Health, University of Southern Denmark, Degnevej 14, 6705 Esbjerg, Denmark

**Keywords:** Qualitative, Field observations, Healthcare, Healthcare professionals, Older people, Clinical care trajectories, Interprofessional, Intersectoral, Person-centered, Habermas

## Abstract

**Background:**

Healthcare services have become more complex, globally and nationally. Denmark is renowned for an advanced and robust healthcare system, aiming at a less fragmented structure. However, challenges within the coordination of care remain. Comprehensive restructures based on marketization and efficiency, e.g. New Public Management (NPM) strategies has gained momentum in Denmark including. Simultaneously, changes to healthcare professionals’ identities have affected the relationship between patients and healthcare professionals, and patient involvement in decision-making was acknowledged as a quality- and safety measure. An understanding of a less linear patient pathway can give rise to conflict in the care practice. Social scientists, including Jürgen Habermas, have highlighted the importance of communication, particularly when shared decision-making models were introduced. Healthcare professionals must simultaneously deliver highly effective services and practice person-centered care. Co-morbidities of older people further complicate healthcare professionals’ practice.

**Aim:**

This study aimed to explore and analyse how healthcare professionals’ interactions and practice influence older peoples’ clinical care trajectory when admitted to an emergency department (ED) and the challenges that emerged.

**Methods:**

This qualitative study arises from a hermeneutical stand within the interpretative paradigm.

Focusing on the healthcare professionals’ interactions and practice we followed the clinical care trajectories of seven older people (aged > 65, receiving daily homecare) acutely hospitalized to the ED. Participant observations were combined with interviews with healthcare professionals involved in the clinical care trajectory. We followed-up with the older person by phone call until four weeks after discharge. The study followed the code of conduct for research integrity and is reported in accordance with the Standards for Reporting Qualitative Research (SRQR) guidelines.

**Results:**

The analysis revealed four themes: 1)“The end justifies the means – ‘I know what is best for you’”, 2)“Basic needs of care overruled by system effectiveness”, 3)“Treatment as a bargain”, and 4)“Healthcare professionals as solo detectives”.

**Conclusion:**

Dissonance between system logics and the goal of person-centered care disturb the healthcare practice and service culture negatively affecting the clinical care trajectory. A practice culture embracing better communication and more person-centered care should be enhanced to improve the quality of care in cross-sectoral trajectories.

## Background

Globally, healthcare and social services have developed and become more complex [1]. Denmark provides an internationally renowned, advanced and admired healthcare system [2]. Despite public funding for most healthcare services [3] and efforts to ensure coherence, many Danish patients still experience inadequate clinical care trajectories across medical specialities and health care sectors [4]. Coordination challenges refer not only to the Danish context but is acknowleged internationally [5].

Over the past decades, the public sector in Denmark has undergone comprehensive reforms, with a shift in responsibility and management [6]. New rationalities won acceptance, and New Public Management (NPM) was introduced based on market structures and optimizing healthcare services’ efficiency [7]. Concurrently, healthcare education programs were modified to accommodate new public sector needs [8], contributing to a change in healthcare professionals’ roles and tasks [9]. Meanwhile, patient’s needs and expectations to patients evolved [10, 11]. A partnership-approach ideology between healthcare providers and patients in e.g. transitional care [12, 13] developed into concrete strategies such as shared decision-making [11, 14]. New perspectives on cooperation between healthcare professionals and patients lead to a renewed emphasis on communication highlighted by many social scientists such as Jürgen Habermas [13]. The introduction of patient involvement altered how the healthcare system regarded patient care pathways [15]. Understanding healthcare treatment as a pathway drew inspiration from industrial processes focusing on quality and efficiency [15]. The concept was applied to healthcare in the 1980s as a reaction to the newly introduced and more case- and diagnosis based financing of care (The Diagnostic Related Group System (DRG)) [16]. Different editions of patient pathways have been modelled, including chain models (high level of predictability, e.g. in elective surgery treatment), hub models (medium level of predictability, e.g. in rehabilitation or internal medicine), and web models (low level of predictability, e.g. in acute care and emergency medicine) taking different levels of predictability of the care processes and level of agreement between healthcare professional teammembers into account [15]. A patient pathway serves to standardize and systematically evaluate the patient-focused care [15]. Placing the patient at the center of care and treatment served to enhance the individual patients’ needs and uniqueness [15], which created a less linear and more complex clinical care pathway [17]. Despite this complexity, patient involvement was popular and included in many organizations’ mission statements [15]. The World Health Organisation (WHO) acknowledged that formal patient participation and engagement contributed to improvement of healthcare services, care culture [18], and patient safety [19]. Thus, adressing the patient’s voice in healthcare was considered a measure of quality [15]. Based on the idea that patients have important resources and needs contributing to a better safety culture [19], tools such as models of ‘shared decision-making’ were encouraged as a central point in treatment [11].

However, formulating new models of care at the organizational level does not automatically guarantee an improvement of patient involvement and personalized treatments at the practical level [10]. Sometimes reality shadows the vision, and person-centered care becomes merely symbolic [12]. It is critical to distinguish between patient- and person-centered care approaches. A patient-centered approach includes the patients’ reasoning for seeking healthcare services compared to a person-centered approach, which has a more holistic approach, including the patient’s history, psychosocial resources, or other factors [10].

On the one hand, healthcare professionals need to deliver efficient, low-cost healthcare services of high quality [20], and on the other hand, they need to provide a person-centered focus in their practice [21]. This gives rise to a dilemma between competing logics [22], within which healthcare professionals need to navigate and manage on a daily basis. Meanwhile, the ever-growing older population with co-morbidities and complex care needs complicates healthcare practice and the delivery of appropriate care pathways [23]. Use of healthcare resources, such as homecare and contacts to the GP, increases with age, and older people are more likely to experience challenges in their clinical care trajectories due to multimorbidities and many transitions between care providers and specialities [24, 25]. These challenges regard the emergency setting as well [23], which might further challenge the care coordination due to acute care needs occurring simultaneously with chronic conditions [26]. Therefore, it is relevant to assess how healthcare professionals interact and provide treatment and care for hospitalized older people focusing on the dilemma between person-centered care and efficiency.

### Aim

This study aimed to explore and analyse how healthcare professionals’^1^ interactions and practice influenced older people’s clinical care trajectories when admitted to an emergency department (ED) and the challenges that emerged.

## Healthcare in Denmark

Denmark provides universal healthcare primarily financed through a tax system, similar to other Scandinavian and Northern European countries. All Danish citizens, approximately 5.8 million, are listed with a general practitioner (GP) [27]. Comprehensive healthcare reform, including decentralization, was introduced in Denmark in 2007 to meet the ageing population’s growing challenges with high usage of costly resources [6]. Thus, healthcare services are currently organized and decentralized at three administrative levels: State (responsible for the overall structure of healthcare), 5 regions (responsible for the hospitals and services provided by self-employed specialists such as GPs and other specialists) and 98 municipalities (responsible for primary prevention and health promotion, including rehabilitation and home care services) [28]. Healthcare agreements describe how the tasks are divided between municipalities and regions to meet the challenges of coordinating care and responsibility between the different administrative levels and care providers [6, 29]. However, the funding structures and governance of older people’s care is complex. As an example, the healthcare reform caused the municipalities to partially co-finance hospital care services, which contribute to economic incentatives [3, 30].

Based on The National Board of Health’s recommendations [31], acute hospitals and establishment of EDs were centralized. Today 21 EDs exist throughout the country admitting all (adult) acute patients, including the older population [32]. The ED intends to secure, effective and fast evaluation of a patient and activate appropriate treatment. If hospitalization exceeds 48 h, the patient is transferred from the ED to another hospital department. Alternatively, the patient can be discharged, receive municipal care, or hospital-at-home care in their private home or nursing home [33, 34].

## Method

Our epistemological approach to the study arose from a hermeneutical starting point within the interpretative paradigm [35]. To capture how healthcare professionals’ practice and interactions influence clinical care trajectory, we used participant observations combined with informal interviews inspired by James Spradley [36]. Field observations can reveal underlying meaning and culture [37], which determines healthcare professionals’ practice.

The study is an individual part of an overall, umbrella project, addressing different but individual perspectives. Other perspectives addressed in the overall umbrella project include among others the perspectives of relatives [38], and older persons’ perspectives to care coordination [25, 39]. Thus, the direct focus on the older persons’ perspectives is not an objective in the present paper. Focus of the present study and paper is on healthcare professionals and their practice.

## Ethical conciderations

The first author observed patients in vulnerable situations, which required substantial, ethical reflections about participation. To manage this, the first author informed every participant verbally and in writing clearly stating the purpose of participating and preparing them for participation until 4 weeks after discharge. The second author, consultant and professor at the ED, functioned as a gatekeeper to the department. However, any trajectories where the second author played an active role were omitted to accommodate the risk of coercion due the authors’ position.

The first author initiated all observations at the ED^2^ attending the morning staff meeting to inform about the study and raise awareness by the staff about the purpose of being present in the department. Approaching potential trajectories was done step-wise. Initial contact was established through the nurse in charge of the patient asking the nurse’ evaluation of whether the older person was eligible for inclusion and capable to give informed consent. Furthermore, accept from the nurse to conduct observations was obtained. Then the first author approached the patient. If they expressed interest, they received verbal and written information about the study and the role of the observer as a researcher and not a nurse. We obtained written consent from all patients, and they were informed that their data would be anonymized, and they could withdraw consent anytime. In accordance with the Helsinki Declaration, participation in the study did not affect treatment or the clinical care trajectories of the participants [40]. We informed all contacted healthcare professionals about the study’s purpose, and the first author’s presence and verbal consent were obtained for every observational situation.

Furthermore, formal consent to conduct the observations in the department was given at the organizational level, aligning concepts of negotiating access to the field [41].

### Being an insider or an outsider

Spradley describes researchers as having a dual role in field observations, as an observer and as part of the field being observed, and is simultaneously an insider and an outsider [36]. In this study, the first author’s role was mostly as ‘observer-participant’, reflecting a low level of participation [36]. However, occasionally this shifted to ‘participant observer’ [37] when patients asked for a helping hand mobilizing, moving or help during a meal. At any level of participation, the researcher was very aware of the older person’s integrity and accept. The duality and shifting of roles became especially apparent because the observer has a clinical background in midwifery (in Denmark, this is an independent education and not a specialization within nursing). Despite many years of obstetric experience in a clinical setting, the observer is not a nurse and has no professional experience treating older people or working within the ED setting. The role as and purpose of a researcher was expressed explicitly to staff, the older persons and their relatives. Furthermore, the researcher wore an identification badge stating the rola as a researcher.

According to Malterud, wearing a uniform affects what type of knowledge and information patients will share with an observer [42]. Initially, the first author (observer) wore a healthcare professional’s uniform as the department nurses during observations serving two purposes: 1) to allow healthcare professionals to identify and engage with the observer during their practice, and 2) requirements from the hospital management in relation to hygienic principles. However, the uniform appeared to distance the observer’s relation with patients, central to the study. Furthemore, wearing a uniform challenged the role as a researcher and level of participation [36] as relatives and other patients approached the first author as a nurse. At those ocations, the researcher stressed the role as a researcher. Thus, in the last three clinical care trajectories, the observer wore private, discrete clothing, which was accepted by the management. The shift in clothing appeared to alter the perception of patients and relatives of the observer as a nurse and enhanced the role as a researcher. This change of perception increased the observer’s legitimacy in accessing the field and neutralized power-dimensions between the patient and the professional.

### Study participants

For this study, we selected clinical care trajectories of seven patients aged 65 years or older at the time of hospitalization. The age cut-off was chosen pragmatically, as it is a commonly used cut-off to distinguish young from older age [43], although we acknowledge that the diversity in perceptions of age is huge. We recruited participants from an ED at one regional hospital which services citizens from four municipalities. To be eligible for the study, the participant had to have allocated daily homecare provided by the municipality. This selection criterion was based upon the presumption that receiving homecare represent a patient group with complex care needs, many care actors involved and a potential for a more challenging clinical care trajectory. Furthermore, participants needed to speak and understand Danish and give informed and written consent. Healthcare professionals, including nurses, hospital physicians, GPs, homecare workers, physio- and occupational therapists involved in the patient case before, during or after hospitalization, were observed or phone-interviewed to explore the interactions in the clinical care trajectory before, during and after hospitalization. The healthcare professionals were employed at either the hospital, municipality, or general practice and Table 1 describes the healthcare professionals contacted or observed concerning a patient’s care trajectory.

**Table 1:** “Overview of observations and interviews”

“Trajectory”, (No. of days hospitalized), [No. of days followed in the study]	Gender, Age (years)	Reason for admission, (Discharged to)	Total observation time^a^, Hours (No. of days), [Observations conducted during day−/evening-shift]^f^	HCPs/staff included in observations^a^	Total time spent on interviews, (No. of contacts)^**b**^	Professions interviewed and follow-ups with the older person, (Duration of each interview),
“Trajectory 1”, (16 days), [44 days]	Female, 85	Fever and chest pain, Pneumonias, Admitted through the out-of-hour medical service, (Own home)	11 h, (4 days), [Day-shift]	Nurses^c^, Physicians, Occupational therapist, Physiotherapist, Porter	2 h, 5 min (11 contacts)	Primary care coordinator (5 min.), Homecare worker 1 (10 min.), Homecare worker 2 (30 min.), GP (General Practitioner) (15 min.), Assessment nurse**** (10 min.), Physiotherapist (30 min.). The older person (10 min., 10 min., 5 min)
“Trajectory 2”, (4 days), [32 days]	Male, 75	Acute abdominal pain, (Own home)	14 h, (2 days), [Day-shift and evening-shift]	Nurses^c^, Physicians, Porter,	1 h, 25 min. (17 contacts)	Nurse*** (10 min.), Primary care manager (15 min.), Primary care coordinator (5 min.), GP (10 min.), Homecare worker (10 min.), The older person (15 min., 15 min., 5 min.)
“Trajectory 3”, (3 days), [39 days]	Male, 70	Fall episodes and functional decline, (Own home)	13 Hours, (2 days), [Day-shift and evening-shift]	Nurses^c^, Physicians, Registrar	1 h, 45 min. (11 contacts)	GP (30 min.), Primary care coordinator (5 min.), Homecare worker (20 min.), Nurse^c^ (25 min.), The older person (15 min., 10 min., 10 min.)
“Trajectory 4”, (2 days), [3 days]	Female, 84	Dehydration and rash, (Died on the 2. Day of hospitalization, thus follow-up with the older woman was not possible)	7 h, (2 days), [Day-shift]	Nurse^cc^ Physicians, Radiologist, Porter,	55 min. (5 contacts)	GP (10 min.), Homecare worker (10 min.), Nurse^c^ (35 min.),
“Trajectory 5”, (17 days), [39 days]	Female, 83	Vomiting and diarrhea, (Own home. Died in the follow-up period. Thus, last follow-up not possible)	14 h, (5 days), [Day-shift and evening-shift]	Nurses, Physicians, Porter, Cleaning and service assistants	2 h, 20 min. (7 contacts)	Homecare worker (25 min.), Nurse^c^(15 min.), GP (10 Min.), Assessment nurse^d^(30 min.), Follow-up via close relative as the older person could not answer the phone (60 min., Text message from relative, −).
“Trajectory 6”, (9 days), [37 days]	Male, 78	Fall episode and dyspnea, (Own home)	8 h, (2 days), [Day-shift and evening-shift]	Nurses^c^, Physicians, Occupational therapist, Physiotherapist, Porter,	50 min. (8 contacts)	Nurses^c^ (5 min.), Homecare worker (15 min.), Discharge coordinator (25 min.), GP (not possible, long-term leave). Follow-up with the older person via homecare worker, (−, 5 min., Text message from homecare worker).
“Trajectory 7”, (2 days, then transferred to another Region for surgery, where observations was not possible), [30 days]	Female, 89	Fall, Transitioned to another Region for surgery, (Rehabilitation center)	7 h, (2 days), [Day-shift]	Nurses^c^, Physicians	Follow-up interview with the older person not possible, as she has no mobile phone. Instead a follow-up visit was conducted at the rehabilitation center, where relevant healthcare professionals where interviewed as well, (1 contact)	Nurse^c^ (20 min.), GP (not possible, unresponsive).
Other*****	–		19 h, (3 days), [Day-shift]	Not specified	Not specified	Not specified
**Total**			**93 h,** **(22 days)**		**9 h 40 min. (60 contacts)**	
**Average**	**81**		13 Hours (3 observation days) pr. trajectory		1 h, 23 min., (9 contacts) pr. Trajectory	

^a^ Observation time refer to the total sum of observations conducted in the representative trajectory at selected times across the number of days counted

^b^Follow-up interviews with the patients and/or relatives are included in the counting of contacts as well as coordinative phone-calls with e.g. secretaries or managers. Coordination activities are not stated in Table 2 as counting interviews though

^c^ Nurses covers hospital nurses, student nurses and/or municipal employed nurses

^d^A municipal employed healthcare professional with a job function to evaluate the older peoples’ needs and determine which municipal services should be allocated

^e^ General field observations conducted in the ED in the day-shift on days where recruiting trajectories was not possible

^f^ Day-shift = before 3 Pm., Evening-shift = after Pm

### Data collection

The first author conducted field observations from January 2019 until May 2019. The majority of observations were made during the daytime as close as possible to the time of admission. However, trajectories were followed in the evening shift as well when relevant. The first author used the patient flow management screens to select potential, relevant patients where the age-criteria was met. We omitted patients marked as ‘isolation’, as healthcare professionals’ practice and interactions might differ in that context.

The first author conducted observations combined with informal interviews [36] of the healthcare professionals involved in the particular clinical care trajectory. When relevant and possible, the first author informally interviewed patients or healthcare professionals. The tracjectories were recruited during the day shift and followed in the evening shift if relevant activities occurred. Furthermore, observations were conducted the following days during the entire period of hospitalization. Follow-ups with healthcare professionals involved before and after hospitalisation were done through phone interviews until 4 weeks after discharge. Follow-up was done with the older persons (or a close relative or caregiver if the person was incapable of answering the phone call) as well one, two and 4 weeks after discharge (For details about the interviews see Table 1). The purpose of these follow-ups was to assess if any coordinating or other relevant activities occurred after discharge. At the last contact with the patient (phone call 4 weeks after discharge), the first author thanked every person for contributing and explicitly expressed, that the observation period ended. We continued to recruit new trajectories until we evaluated, saturation was met, which resulted in seven clinical care trajectories to be included in the study.

The first author took handwritten field notes during the observation time and conducted transcriptions at the end of the same day or the following day. During the in-situ observations of patients and healthcare professionals, the first author used jottings [44] and later withdrew to a private place to transcribe these jottings to field notes after each event. Furthermore, reflective notes were kept when relevant – aligning a reflective diary.

To explore the care trajectory before and after hospitalization, the first author conducted unstructured phone interviews with all, relevant healthcare professionals involved in the primary care before and after hospitalization, including GP’s. During the phone interviews, the first author conducted handwritten notes, which where converted into more formal field notes afterwards. All field notes, including observational notes and interview notes, were transcribed.

### Analysis

**Fig. 1:**
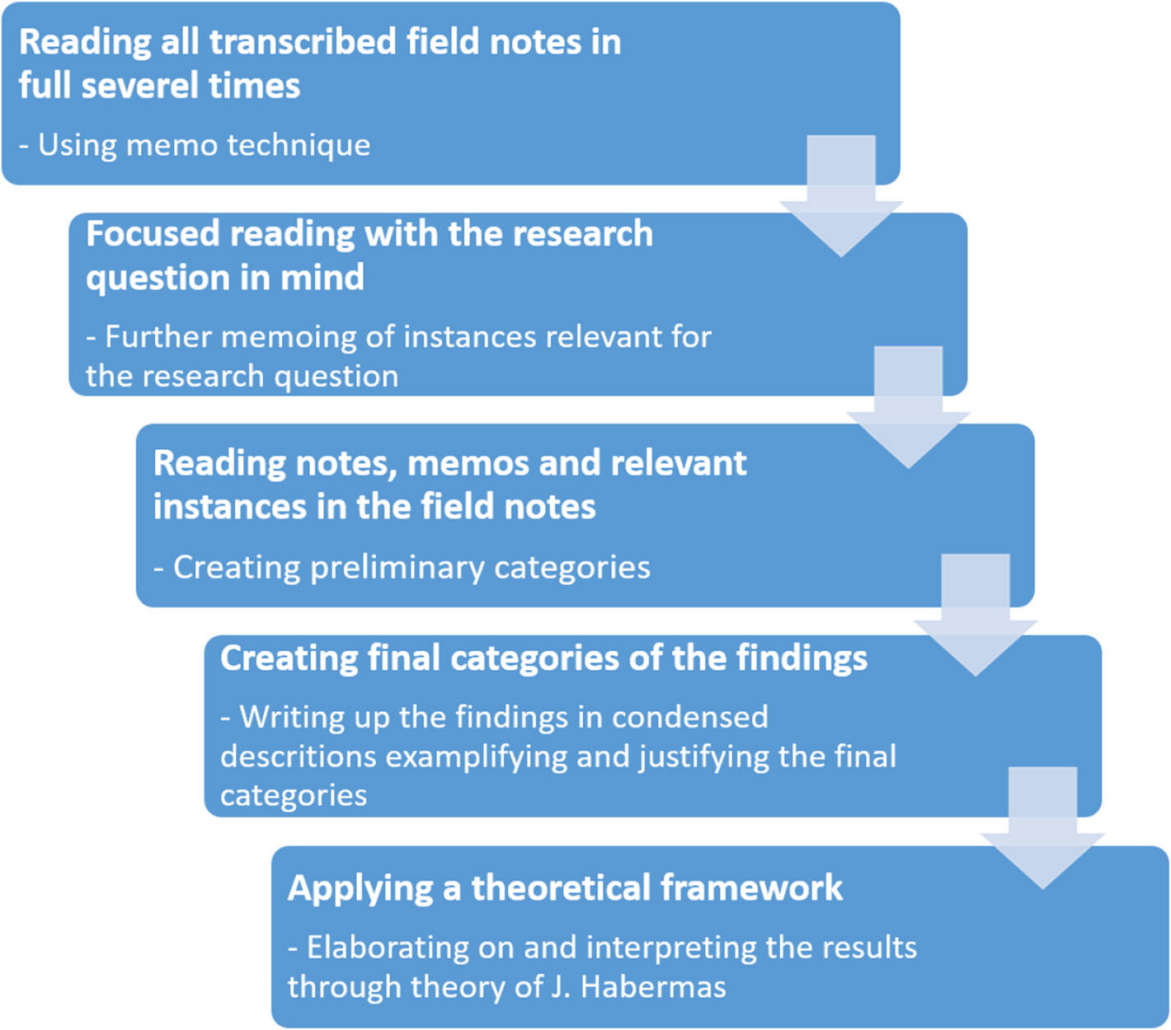
“Illustration of the analysis process”

The approach to analysis was inspired by the work of A. B. Andersen and colleges [34]. Andersen et al. drew on Blumer’s methods of letting the empirical data correspond to and wake the researcher’s interest [34, 45] (See Fig. 1 below).

The analysis procedure was initiated by reading all field notes (both from observations and interviews) multiple times using the memo technique [44]. Then the authors continued reading one trajectory after another independendly looking for empirical episodes of specific interest in relation to the study’s aim. To enhance credibility and rigour, all authors discussed the trajectory events, confined and condensed them, and created representative categories justifying the findings. (See Table 2 below). The authors benefit from having different backgrounds and employments providing different perspectives to the analysis process. The first author has a background within midwifery as mentioned and public health. The other authors are all employed as researchers either at the university or hospital and represent backrounds within sociology, farmaceutics, general practice and internal medicine. Two are male authors.

**Table 2:** “Examples of the data analysis”

Instance examples	Memo examples	Examples of preliminary categories	Extract examples of condensed descriptions	Final category
The older woman (in trajectory 1) asks for water in a sip-cup after transferred to the geriatric department. Her wish is expressed more than once but overruled by a nursing practice to prevent swallow failure and pneumonia risk	The woman used a sip-cup at the ED. In the geriatric department her wish is ignored. She is offered a straw instead to prevent swallow failure. Practice seems conflicting with patient needs or wishes.	Conflict between practice and patients’ needs	*Due to a hemiparesis, the older woman uses a sip cup (with a spouted lid) to drink without assistance. Despite asking for a sip cup at the geriatric department, the nurse comes with a regular drinking glass. The nurse explains that drinking from a sip cup can contribute to failure to swallow correctly, increasing the risk of pneumonia. To compromise, the nurse brings the older woman a straw to use. However, this is also impossible for the woman to use.*	The end justifies the means – “*I know what is best for you*”
The older woman (in trajectory 5) was admitted after several days of severe vomiting and diarrhea. She has not been eating or drinking sufficiently for days. Two small juice boxes has been placed besides her, but she is not able to drink due to the straight straw. She was neither offered lunch.	I wonder what role basic care has in the older woman’s CCT, and if basic motivation to eat and drink and better preconditions (As e.g. an appropriate straw) would have benefited the woman’s CCT	Basic care needs	*I ask if she would like something to drink.* *She says, “Yes, but the apple juice is empty, and I don’t like orange juice”.* *As I lift the apple juice, I realize that it is half-full, but due to the straight straw, impossible for her to drink, giving the impression that it is empty. After locating a bendable straw, she can drink by herself. As she has not eaten any lunch and it’s past noon, I ask her, if she has ordered anything.* *“No”, she says, “I don’t feel like eating anything”.* *“Well, what about a small soup then”, I suggest.* *“Well, I think I can eat that”, the older woman replies.*	Basic needs of care overruled by system effectiveness
There are different perceptions of, what the older person (in Trajectory 2) suffers and what treatment is most appropriate. The physicians argue whether or not he is a cardiac patient.	Organisational structures and power clashes between professions and entities affect the care planning in the CCTs	Care coordination across settings	*Later, the nurse calls the medical department to arrange the transfer to continue treatment for heart failure. The nurse at the medical department says to the ED nurse that she will try to arrange a ‘trade’ by moving one patient from the cardiology department to the medical department, to be able to transfer the older person directly to the cardiology department. After hanging up the phone, the nurse turns to me and says: “Well, now my patient is involved as a bargaining chip to receive the best treatment.*	Treatment as a bargain
The older person (in Trajectory 3) is admitted by his GP for thorough investigation after fall episodes in the home and general declining level of function. The GP is familiar with his use of alcohol.	People who are not able to be proper carriers of information’ is a challenge for the care coordination, responsibility is mis-placed unintendedly with the pt.	Abrupted care distorted by the pt’s/person’s perspectives	*During the person’s hospital stay, he is examined by several physicians to find an explanation for his fall episodes. Every time he is approached by a medical professional, he has an alternative explanation or reveals a new place of pain or raises an additional problem. This results in, every physician having a new focus for treatment.*	Healthcare professionals as solo-detectives

Finally, to explore the possible reasons behind our findings, we applied the theoretical framework of Jürgen Habermas’ ‘Lifeworld’ and ‘System’ [46, 47], as we believe that his perspectives on ‘system’ and ‘lifeworld’ have certain explanatory power in elaboration of the results insinuating dissonance between healthcare service cultures and the rhetoric of person-centered care approach.

## Results

The analysis of the practice and interactions of the healthcare professionals in this study revealed four main themes: 1) “The end justifies the means – ‘I know what is best for you’”, 2) “Basic needs of care overruled by system effectiveness”, 3) “Treatment as a bargain”, and 4) “Healthcare professionals as solo detectives”. The results insinuate that person-centered care is limited. Other logics such as a paternalistic approach based on a misunderstood sense of care seemed to dominate older peoples’ clinical care trajectories.

### The end justifies the means – “I know what is best for you”

Our results show that healthcare professionals may unintentionally practice inappropriate care dissonating person-centered care approaches. The following examples demonstrate how healthcare professionals misinterpret the needs and expectations of their patients.

***“Woman, 83” (Trajectory 5):***

*A woman was admitted to the ED eight days ago due to nausea, vomiting and insufficient eating and drinking over several weeks. On the second day of her hospital stay, she was transferred to the geriatric department for further assessment and treatment. I visit the woman in the geriatric department just before noon. The door to her room is open, and from the hallway, I can see her sitting in an armchair bent forward with a tired look in her eyes. She observes the healthcare professionals walking back and forth in the hallway and tries to catch their attention. When she notices me, she looks at me and says:*

*“I need to go back to bed”.*

*I suggest that she calls her nurse by pulling the call cord.*

*She asks, “Where is that? [the call cord]”, and I show her, that the cord is attached to the armchair. I help her pull the call cord.*

*Eventually, a nurse in a white uniform approaches. The nurse stands in the doorway, leaning her right shoulder against the doorframe. The nurse looks at me inquiringly and turns towards the older woman, waiting expectantly. The woman says nothing. I explain to the nurse that the woman expressed a need to come back to bed, and she replies:*

*“Yes, but she’s only just got up, I think”. The nurse remains standing in the doorway, “I will tell the other nurse”, then leaves in the direction of the nurses’ office, located opposite to patient’s room.*

*A short while later we can hear the first nurse say to a colleague: “Just as I thought”. Then the nurse re-enters the doorway and addresses the woman: “I have told your nurse, but you have just come out of your bed. The other nurse will come in a minute”.*

*The woman raises her arm as if trying to prevent the nurse from leaving and responds, “No, that’s not true”.*

*The nurse repeats oneself “Your nurse will be here in a minute”.*

*The woman asks: “But when is a minute?”.*

*The nurse says “Well, I don’t know. The other nurse is busy on the computer”. The nurse then leaves.*

*The woman looks at me and shakes her head: “They say in a minute, and then it takes half an hour. It’s not easy growing old!...”*

In this trajectory, the woman’s needs are overruled by the practice and interactions of the two nurses. The apparent basis for their actions is a belief that sitting in the armchair is best for her, who would otherwise be lying in bed all day. However, it was against the clearly expressed wishes of her and without her acceptance. The two nurses’ interactions dominate the care practice, and neither reacts to the woman’s clearly expressed needs.

A similar example is Trajectory 6, where the older man’s wishes for his hospital stay are ignored and he is transferred to the medical department against his will as the situation below demonstrates:

***“Male person, 78” (Trajectory 6):***

*The man was acutely hospitalized after a fall-episode in his home. He is examined for a leg fracture in the ED, and despite no fracture, the hospital staff suspect he has pneumonia due to shortness of breath. he is admitted to the ED for further examination and evaluation. A student nurse working under supervision of a registered nurse is involved in the care (The registered nurse is not present with the older person throughout the observations). His infection count (Creatinine Reactive Protein) increases and he requires increased oxygen supply, something with which he is familiar at home due to his COPD (Chronic Obstructive Pulmonary Disease). Two physicians, a younger and a more experienced physician, discuss best treatment options. They decide if he can be mobilized and swallow antibiotic tablets, he can be discharged and continue antibiotic treatment in his home environment. His ability to swallow is evaluated and no concerns are identified. Then two therapists (Physiotherapist and occupational therapist) motivate, help, and mobilize him from the hospital bed to an armchair in the same room. However, he refuses to cooperate. He is mobilized to an upright sitting position on the edge of his bed. The therapists conclude that he meets the physicians’ criteria for discharge. The student nurse approaches the therapists in his room and follows them out into the hall to the staff office. Whilst walking, the student nurse expresses the opinion that discharging him is not optimal as he is not drinking or eating sufficiently. The student nurse states that a transfer to the medical department would improve his situation. At the office, the two therapists discuss their considerations with the nurse supervisor. The therapists question whether he refused to be mobilized due to anxiety of falling or lack of ability to walk. The student nurse continues to argue that he would be better off transferred to the medical department, which the supervising RN nurse supports based on the student nurse’s argumentation. The student nurse approaches the younger physician in the hall and states that it was impossible to mobilize the older man. The physician accepts that he ought to be transferred to the medical department the same day.*

*Later, I approach the older man to ask what he thinks of the plan. He clearly expresses that he is very unsatisfied with not being discharged. His home care worker, who is interviewed by telephone after his discharge, confirms his dissatisfaction. The homecare worker explains that he clearly expressed that hospitalization was not a good experience as the hospital staff were too busy and did not listen. After his discharge, he is in a similar physical state as he was when he was first hospitalized but does not feel well. Whilst in hospital, he did not receive his usual laxatives. As a result, he was very constipated after discharge.*

Presumably, the student nurse is convinced that transferring the older person to the medical department is best for him. This belief affects the nurse’s practice in a way that places uncertainty on the physiotherapists’ evaluation and misleads the younger physician. Thus, the older man is transferred to the medical department against his will. Furthermore, he experiences discomfort during and after hospitalization due to lack of treatment with laxatives. In this situation, even though the student nurse had the best intentions, the older man ends up with inferior treatment, constipation and an experience of not being heard.

Good intentions of countering harm are not necessarily equivalent to complying with the needs and wishes of the patients. A paternalistic agenda can distort the foundation for patient-involvement with a negative outcome for the patient, as exemplified below:

***“Woman, 84” (Trajectory 4):***

*The woman had been admitted three weeks earlier with (congestive) heart failure. Since then she has lost 8 kg and is dehydrated with deranged electrolytes, and she is now hospitalized again. The physician wants to measure her urine output but leaves it up to her to decide on whether to use a toilet chair or have a catheter applied. She clearly states that she would prefer to use the toilet chair. The physician approaches the nurse at the office to brush up on the care plan. The nurse questions the physicians’ decision to allow the woman to use a toilet chair. She asks the physician directly:*

*“Is she aware, that her kidneys might fail if she does not have the catheter [Implied that precise measurement of urine output contributes to proper treatment decision]? Shouldn’t I have a chat with her?”.*

*“Yes, talk her into it”, the physician responds.*

*The nurse finds the woman in her room, sleeping. She briefly explains that it would be best for her to have a catheter applied.*

*“Hmm”, she says and shrugs.*

*The nurse leaves the room and passes the task of the catheter to another nurse colleague; “Please do it as soon as possible, because she is not that keen on it”, she says to her colleague.*

*Both nurses enter the woman’s room, and the first nurse explains that the colleague will apply the catheter.*

*The woman asks: “Am I supposed to have a catheter applied?”,*

*and the nurse replies “Yes, you just said you would”.*

*The woman shrugs again.*

*After applying the catheter, the nurse says in a comforting tone to the woman:*

*“Well, it’s all over now”,*

*and the woman replies: “But it’s not having a catheter applied [that I don’t like], it’s having to have it …”*

The woman’s condition is serious, and the choices of treatment and efforts have consequences, which may explain how the physician and the nurses justify their approach to her care. However, the groundwork for her consent is questionable. Her tacit acceptance of the application of the catheter can be seen as a result of her ill and exhausted state.

Another example of how a care provision standards can weaken a patient’s integrity is expressed in Trajectory 1. In the example below, the use of a sip cup disturbs person-centered care:

***“Woman, 85”, (Trajectory 1):***

*The woman was admitted to the ED due to high fever, chest pain and dyspnea (pneumonia). On the second day of hospitalization, I meet the woman in the ED before she transfers to the geriatric department where treatment is continued. I follow her to the geriatric department. After an earlier stroke, she has a paresis. Due to her hemiparesis, she uses a sip cup (with a spouted lid) to drink without assistance. Despite asking for a sip cup at the geriatric department, the nurse comes with a regular drinking glass. The nurse explains that drinking from a sip cup can contribute to failure to swallow correctly, increasing the risk of pneumonia. To compromise, the nurse brings her a straw to use. However, this is also impossible for her to use.*

Here, the woman clearly expresses her needs and offers staff a solution, as the sip cup enables her to drink independently. However, the nurse does not meet her wishes, probably due to a concern about increasing risk of pneumonia, and she ignores woman’s request. As per se, the nurse’s attempt to counter actions of harm determines the practice and outweigh the patient’s need to maintain independence.

### Basic needs of care overruled by system effectiveness

Throughout the observations, the interactions between the healthcare staff and patients were in many situations limited, deficient, or lacking. These interactions were suboptimal often due to high workflow, so patients were left alone for considerable amounts of time. Patients had the opportunity to call for assistance by using the call cord. In the trajectory below, the young nurse has had several extra workdays due to sickness leave amongst her colleagues. In this trajectory, we meet the older woman the day after she was admitted to the ED:

***“Woman, 83” (Trajectory 5):***

*The woman admitted due to several days of severe vomiting and diarrhea, and apparently, she has not been eating or drinking sufficiently for several days.*

*Before entering the room the first time, I approach the nurse who says to me:*

*“She [referring to the older woman] prefers to vomit while you are present in the room, but she doesn’t vomit. I have been there several times, and nothing happens, she just spits a bit. My guess is that it’s treatment with fluids for a day and then home again [to her own home]”.*

*It is late morning. I see the woman lying in bed and has no venous access to administer any type of intravenous therapy. She is a skinny and pale woman with tired eyes, but she still smiles when I approach. I see two small juice boxes on her bedside table, one with apple and the other with orange juice. Stressing the importance of drinking, I ask if she would like something to drink.*

*She says, “Yes, but the apple juice is empty, and I don’t like orange juice”.*

*As I lift the apple juice, I realize that it is half-full, but due to the straight straw, impossible for her to drink, giving the impression that it is empty. After locating a bendable straw, she can drink by herself. As she has not eaten any lunch and it’s past noon, I ask her, if she has ordered anything.*

*“No”, she says, “I don’t feel like eating anything”.*

*“Well, what about a small soup then”, I suggest.*

*“Well, I think I can eat that”, she replies.*

*When I leave the room to let the nurse know, that she is ready to order soup, she turns to me and with lifted eyebrows says:*

*“Not everyone should be a nurse! Well, can you hear me, said the blind person to the deaf person” as a statement describing her experience and treatment.*

The woman seems nauseous and expresses dis-like of eating or drinking. However, the observer manages to motivate her to drink by solving her challenge with the straw, showing interest and cheering her up by being present with her. The woman seems unsatisfied with the care. This nurse has accepted extra working hours and is making an extra contribution to the ED. It seems like the nurse finds the older woman’s care demanding, which may be a reaction to the workload or high patient turnover, typical in an ED. However, the nurse overlooks the woman’s needs to be encouraged to eat and drink, and the nurse’s interactions and care and treatment seems marked by conflict.

The next day the woman is transferred to the geriatric department for 2 weeks. She dies, 2 weeks after discharge, in her own home.

The following trajectory, where we meet an older man in the early afternoon in a situation around receiving an enema, describes the workflow, work culture, structure and organization of the ED and how these factors can affect care and treatment:

***“Male, 75” (Trajectory 2):****was admitted with abdomen and chest pain. Seven years ago, he had had back surgery after an accident. Due to the surgery, he has a urinary catheter and can only walk a few steps with a Zimmer walker and a helper such as his wife.*

*Despite being admitted to the surgical section of the ED, examinations at the hospital showed that his abdominal pain was due to severe constipation. Part of his treatment for constipation is having an enema. While the nurse organises the enema, The wife (who is visiting) and I leave the room. The nurse tells the older man and his wife that the man can call for assistance to use the toilet chair by using the call cord. The wife waits in a lounge area nearby, and after a short chat, I leave her in privacy. After a while, I return but find her still waiting outside her husband’s room. She says:*

*“I don’t know if the nurse is with him, because he has called ages ago [a red lamp is marked outside the room], but I haven’t seen the nurse yet”.*

*We enter the room and cannot find any nurse. Instead, we find the older man to have emptied his bowels in his bed and is now sleeping. He had not been assisted onto the toilet chair. The wife seems upset. I look at my watch and realize that there has been a change-of-shift and wonder if that could explain the lack of reaction to his cord call. I share my thoughts with the wife and suggest they alert the nursing staff again. Later, I ask the nurse who took over after the day shift about the treatment status. With enthusiasm and positivity, she explains that he has successfully emptied his bowels, then has been washed and cleaned up unproblematically with a new pair of disposable briefs.*

The reaction from the nurse to the situation that the older man was not assisted to the toilet chair could insinuate that the nurse approaches the situation as a regular nursing task. The nurse does not seem to notice or realize any conflict with the situation. However, the nurse’s positive description of the task could be a defensive mechanism after not responding to his call. The shift between day and evening does not overlap and may explain why there is no interaction or coordination between the two nurses. Unfortunately, the situation leaves the older man and his wife frustrated and distrustful.

Trust is an essential factor for the experiences of patients and their relatives, and information builds trust. The following example shows how a lack of information can contribute to distrust and skepticism with treatment decisions:

***“Woman, 85”, (Trajectory 1)*** *:*

*During the hospital stay, the woman is examined by a cardiologist who gives the all-clear concerning her heart. However, her home care worker reveals in an interview with me that the woman’s daughter discovered unfamiliar medicine (nitroglycerin) when unpacking. The daughter asks the homecare worker whether she knows anything about the unfamiliar medication. One does not. Neither does the older woman. However, the home care worker remarks that the cardiologist did not find anything suspect in the heart examination and then also wonders about the unfamiliar medication.*

In the woman’s situation, the hospital has prescribed and handed-out medication without informing relatives or colleagues in primary care sufficiently. The homecare worker reflects that the woman’s heart examination was clear and has no explanation for this medication. Thus, it creates distrust and skepticism of the healthcare system for the daughter and the homecare worker.

### Treatment as a bargain

There is a tendency for clinical care trajectories to be dominated by negotiations and underlying power battles. In Trajectory 2, this becomes apparent due to the person’s complicated and ambiguous symptoms which clash with treatment and responsibilities of physician’s specialties in the ED.

***“Man, 75”, (Trajectory 2):***

*After it was apparent that the man’s abdominal pain was caused by constipation, he was evaluated for dyspnea. The responsibility for his treatment was passed from surgeons to cardiologists who examined him for heart failure. On the second day of his hospitalization, his nurse says:*

*“Well, now he has belonged to every specialty here in the ED except gynecological. First, he was a surgical patient, then a cardiac patient and now a medical patient.” The nurse does not think he has heart failure but thinks his shortness of breath is a learned behavior after his back surgery. Therefore, the nurse arranges a consultation with the physiotherapist from the department. They examine him together and agree that he is not suffering shortness of breath. The nurse wants to discuss the findings with the physician at the hospital round but has to leave the room, stating that she will return shortly. When she returns, the physician has completed ward rounds and left.*

*I find the physician in his office and ask him about his role in the treatment:*

*“Well, I have just made a plan. The cardiologists will not take the patient. I have presented the case clearly, the cardiologist agrees to the plan, but will not be in charge of their own patient.”*

*When I ask the physician about his reflections, he replies:*

*“Because they are very tied-up...(They pass on responsibility) To prevent them from drowning in these sorts of patients. The patient will get the same treatment, but with us in charge. As he turns away, I point out that his contribution will be anonymized. But he turns to me and says:*

*“You can use my name AND state that this patient belongs to cardiology!”*

*Later, the nurse calls the medical department to arrange the man’s transfer to continue treatment for heart failure. The nurse at the medical department says to the ED nurse that she will try to arrange a ‘trade’ by moving one patient from the cardiology department to the medical department, to be able to transfer him directly to the cardiology department. After hanging up the phone, the nurse turns to me and says: “Well, now my patient is involved as a bargaining chip to receive the best treatment. According to my ethics, I think it is a bit (She puts her hand to the heart…)”, leaving the impression, that she thinks the care process is highly inappropriate.*

The organizational structures facilitate power imbalances between physician specialties but also between professions. Instead of inter-professional collaboration for optimal treatment, the agenda seems to be demonstrations of power or victory. The ED nurse attempted a thorough evaluation with the cooperation of other professional colleagues, e.g. physiotherapists. However, the treating physician is eager to do battle with the cardiologists. The phycisian does not respond to or overlooks important information from the nurse. Trajectory 2 furthermore reflects the hospital’s organizational structures and power clashes. These structures are continuous and affect the man’s opportunity for transfer to another department. The nurses end up using bargaining to create a flexible and appropriate solution for him.

Negotiation was seen as a common approach used in other situations. In Trajectory 1, two physicians have different approaches and agendas for medication, and they implicitly use their range and level of experience in the underlying negotiation, which the older woman was neither part of or able to affect.

***“Woman, 85”, (Trajectory 1):***

*A registrar receives the older woman at the Geriatric department right after transfer from the ED. Whilst going through her journal, the registrar notices a very high (beyond recommended) daily use of morphine. The registrar asks the woman about the pain she experiences and suggests finding a better solution. She is reluctant, stating that she needs the morphine. The registrar assures her that he will find an alternative and slowly taper off the morphine. She accepts this course of treatment.*

*At the office, the registrar rings the woman’s GP to understand the reasons behind the high use of morphine and to discuss a better treatment plan for the woman. Unfortunately, the GP is unavailable. The following day the registrar is not at work, and another, more experienced physician is in charge of the treatment. The physician has reduced and substituted woman’s use of morphine with an alternative. This change of treatment plan occurs without any dialogue or collaboration with the GP. When I later ask the registrar about the process, and if the experienced physician have tried contacting the GP again, the registrar says ‘no’ and explains, that the colleague is more experienced and might not have had the need to discuss medication change with the prescribing GP.*

In the above example, the negotiation that affects the trajectory is the level of professional experience. The registrar initially evaluates that a discussion with the woman’s GP could be beneficial. However, the more experienced physician does not share this view and independently changes the medication. This practice demonstrates underlying and hidden power structures that affect physicians’ interactions and, ultimately, the woman’s clinical care trajectory.

### Healthcare professionals as solo-detectives

In other situations, the patient is approached with a very narrow focus, although the patient’s issues are complex and multi-faceted. As an example, Trajectory 4 demonstrates how one single issue can be more prominent than a holistic, person-centered focus:

***“Woman 84”, (Trajectory 4)****:*

*Though the woman’s hospitalization focuses on dehydration and abnormal fluid count, she has a rash, which the physician is keen to examine. I witness the physician’s initial examination of the woman. The physician asks her very systematically about her medication using medical terminology. One question follows the other without time for her to respond. It comes to a point where she takes a breath and says quietly:*

*“I don’t know what all that is”.*

*The physician leaves the room without any explanation, and a resigned older woman glances at me, “shrugs” and says:*

*“Hmm, I think it would be better if I’d stayed home and kept quiet…”.*

*After a short while, the physician re-enters the room purposefully, and a colleague physician follows him. The first physician asks the colleague to evaluate whether the rash is scabies. The colleague rejects a scabies diagnosis, and the physician then replies:*

*“Okay, then it must be caused by her medication!”.*

The physician’s focus on solving the mystery of the woman’s rash overshadows the more severe condition she is suffering. The physician attempts to identify the reasons for the rash very systematically using all the right medical terminology. In his eagerness, he does not notice, that The woman seems overwhelmed by the medical terminology and his practice and actions seem incompatible with her needs.

In the example, the story demonstrates how a patient can be lost in a system’s efficiency dominated by a mechanical, professional approach to patient challenges. Increased or exaggerated awareness of a single challenge for a patient can result in less focus on the human as a whole person.

Person-centered care is also challenging for older patients with multi-faceted, complex problems or co-morbidities, as was clear in the following example:

***“Man, 70”, (Trajectory 3):***

*An older man aged 70 years is hospitalized for three days due to a series of falls in his home. The GP referral is thorough and states a request to investigate the complexities of his situation, including issues such as compliance problems with his medication, alcohol use, and deep venous thrombosis. However, the hospital plan is somewhat simplified and reported as ‘cardiac rhythm assessment’.*

*During his hospital stay, he is examined by several physicians to find an explanation for his fall episodes. Every time, he is approached by a medical professional, he has an alternative explanation or reveals a new place of pain or raises an additional problem. This results in, every physician having a new focus for treatment. When he is finally discharged, the physician decides to discontinue his prostate treatment with the theory, that dizziness (a side effect of the medication) is causing his fall episodes. Furthermore, he is a heavy smoker, and complains among other problems about a blocked nose while hospitalized. At the time of discharge, he receives a prescription for a Nasal decongestion spray.*

*I interview his GP, primary nurse and home care worker individually, and they are all aware of the amount of alcohol he drinks. The home care worker estimates, that he drinks about 3 liters of wine daily, as she carries out his garbage. The primary care nurse and GP both suspect that he has alcohol-related dementia, explaining his difficulties giving clear descriptions and reasons.*

*In addition, the home care worker has observed that after his prostate medicine is discontinued, he has wet the bed each night. Despite, the precise allocation of help he is entitled to (in which laundry is not included), the home care worker chooses to assist him with his extra washing. As incontinence has not yet been evaluated as a problem, there is no allocation of disposable briefs for him. Thus, the hospital’s solution to his complex situation based on the GP's referral is limited to a prescription for nose spray and a change in medication resulting in additional problems.*

The man’s condition complicates his ability to explain and describe his own needs, as he may be limited by long-term and excessive alcohol use. Complicating matters is the over simplification and condensation of the referral from his GP. With limited coordination across health sectors and healthcare providers, patients must be able to communicate clearly, something which he is incapable of. Thus, his clinical care trajectory is fragmented and disrupted. This study could point to a tendency, that the healthcare system is not adaptive to vulnerable patients with questionable communication capabilities, and responsibility is still placed, unreasonably, with these patients.

## Discussion

Different reformative changes have been introduced in attempt to accomplish better care coordination across health sectors. Lately in Denmark a more comprehensive reform (The Healthcare reform, 2007) included changes in the administrative levels (State, regions and municipalities) contributing to a decentralization of the healthcare system where tasks and greater responsibility was sled to the municipalities followed by a lowering of the mid-level (referring to the regions responsible for the hospitals) [6]. Alongside, new management rationalities, often reffered to as New Public Management (NPM), gained entrance, which was introduced streams of privatization and marketization of the healthcare and public sector [7].. Our study shows that patient involvement and mutual decision-making tends to be limited in the Danish healthcare system. Despite the nursing staff’s good intentions, patient care is often focused on countering harm rather than taking individual needs into account, e.g. when the older man in Trajectory 6 is transferred instead of discharged, and the woman in Trajectory 5 is mobilized against her will. As a result of e.g. time constraints and limited resources, healthcare professionals care for patients without properly engaging and communicating with them exemplified in Trajectory 4 where the physician practices an inappropriate use of professional terminology with the older woman.. Other logics may guide the approaches to care in the trajectory of the clinical care pathways. Often the agenda of professionals, to do what they perceive is the best thing for the patient, may differ to the wishes of patients and/or relatives. This neglect to person-centered care is exemplified in Trajectory 1 where the older woman is not allowed a sip cup.

We suggest that healthcare professionals’ navigation between these conflicting agendas represent a clash between service cultures and care provision (‘New manegerialism’) and the intention to act person-centered This clash can contribute to explaining the problems with delivering coherent and holistic oriented clinical care trajectories as the healthcare practice is affected by e.g. structural demands and organizational procedures, which inhibit the potential of a holistic person-centered care approach.. On the one hand, the healthcare system demands effectiveness, and on the other, it expects increased patient involvement and individual care plans. Thus, Habermas’ theory and concepts of ‘lifeworld’ and ‘system’ [46, 48, 49] are useful in partially explaining the conflicting relationship between the healthcare system, the healthcare providers who are part of the system, and their patients.

### Applying a Habermas approach to the results

Habermas uses the term ‘Lifeworld’ that represent the social world of individuals referring to interactions in the private sphere e.g. with family or friends, or interactions with others in the public domain. According to Habermas, the ‘System’ refers to the economy state and the power of the steering media, that may intrude onlifeworlds in unaccountabel ways (colonization) [46, 50, 51].

The system, of which the healthcare system and HCPs are part of, is often dominated by economic- and market-thinking, this colonizes our lifeworld through suppressive, albeit unconscious, ideologies [46, 49]. When applying Habermas’ concepts of ‘lifeworld’ and ‘system’, the healthcare practice and coordination activities are affected by the ‘system’. However, HCPs’ care of patients may also be influenced when their own lifeworlds are bought into their practice.

Habermas describes the term ‘power’ in social practice and interaction [48]. He suggests that a solution to the imbalance of power between the system and lifeworld, which applied to our study could be clash between the healthcare practice (care provision) and patient- or person-centered focus, often lies within rational dialogue and interactions between people (Healthcare professionals representing the system and the older people/patients) giving the potential for freedom and mutual understanding [13]. The dialogue can shed light upon the potential colonization of the healthcare practice and contribute to a better understanding between healthcare professional and patient resulting in greater potential for person-centered care.

### A dominating system with market logics

Based on our study we suggest that the limited patient involvement and lack of focus on patients’ individual needs, as revealed in our analysis (e.g. Trajectory 5 and 6), could be a result of a dissonance between a dominating system logic where efficiency and checklist procedures compete with patient- or person-focused nursing care. In Habermas’ terminology, it could be seen as a colonization of the healthcare practice. However, strict time- and resource-constraints are not new issues within the public sector. The modernization of the public sector has been on-going since the 1980s striving to increase public service quality and efficiency [8].

The NPM approach causes competition across the public and the private sectors [8] as seen by private suppliers’ eligibility to provide homecare to Danish citizens since 2002 [52]. In a study from 2014, care as a market activity was discussed [53]. The study highlighted that market thinking directly affects care provision and thus indirectly the relationship between the healthcare professional and the older person receiving homecare, similar to a service provider and a customer’s role [53].. This negotiation approach to care came into focus in our study of Trajectory 2, where the two nurses refer to the patient’s transition to the cardiology department as a bargain, and the power struggle between his physicians and the relevant specialties is observed. In Trajectory 1, negotiation was seen in relation to the patient’s medication. The clinical care trajectory in Trajectory 4 exemplifies how communication can be practiced less appropriately with a tendency towards a mechanical approach.

### Developing new professional identities

Meanwhile, implementation of NPM required documentation and performance-measurement [52], referred to by Michael Power as the ‘Audit-society [54]. Furthermore, evidence-based medicine gained momentum and treatment became more specialised [8, 9]. These new demands encompassed new tasks and practices for healthcare professionals leaving less direct contact with patients and relatives. The outcome of system efficiency is seen in Trajectory 2, where the physician finishes his hospital rounds so timely that the nurse has no chance to discuss her reflections with him.

It has been argued that the development of nurse’s professionalism is an action that arose from nurses’ motivation to gain more status [13, 55]. However, applying Habermas [46], the perception could be, that the altering within the professional profiles occur as a colonization from the system, which affects healthcare professionals’ lifeworld. Thereby as practices and procedures change, healthcare professionals will slowly view their roles in clinical care trajectories differently. The new professionalism embracing increased patient demands, involvement, and mutual decision-making affects the relationship and power division between the professional and the patient. Several scholars, such as Talcott Parsons, have described how the roles of and relationship between healthcare professionals and patients are distorted by power division [8]. The more paternalistic approach to care, revealed in our study (e.g. Trajectory 1 and 5), can be seen as a misuse of power or a tendency to work from an understanding of the professional as the professional expert, as described by Parsons and others in the 1970s [56]. This approach has been criticized as inappropriate as it appears to neglect the lifeworld of the patients. According to Habermas’ theory, it is crucial to raise awareness of power relations through a reflective process to avoid increasing of the power gap between healthcare professionals and patients [48]. The potential for mutual understanding and decision-making are formed within this relationship and partnership between HCPs and patients. Thus, communication and equal dialogue between patients and healthcare professionals are essential to improve quality and support person-centered care within the clinical care trajectory.

However, our study insinuates a paradox between striving to involve patients and the competing requirements of increasing efficiency and care provision. This is similar to findings in a recent study adressing pain care provision and practice culture that clash with the patient’s perspective [57]. The tendency to limit communication with patients and relatives may contribute to a less person-centered care approach. It signals that an unbalanced focus on system effectiveness facilitates a dominating market logic, inappropriate for person-centered care. Sine Lehn describes a healthcare practice affected by system logic. Lehn highlights new challenges for healthcare professionals’ work practice that can result from system and organization changes [58]. Without a practice culture that embraces person-centered care and communication, there is a risk, that market logics and scarce resources will dominate the healthcare profession and reduce care to trade. In that case, there is a tendency to evaluate patients more as consumers or customers requiring a service.

### Complexities of the ‘lifeworld’ and implications for practice

Our study suggests that healthcare practice is significantly colonized and is challenged by system effectiveness demonstrated in the trajectories described earlier. However, the healthcare system is complex. Even though healthcare professionals’ practice is based upon procedures and structures, their lifeworld is individual and affected at different levels. To practice more patient- or person-centered care requires healthcare professionals to activate their own lifeworld to engage in the individual patient’s lifeworld whilst communicating and acting at a professional level. Healthcare professionals are not only professionals delivering healthcare services, they represent the lifeworld as well. They view the world differently and have individual lifeworlds, which contribute to the complexity in professional interactions.

The study indicates that organizational care plans and care provision should not merely be based on market logics and NPM thinking. There is a need to emphasize the care approach prioritizing person-centered care and relatives’ involvement, to achieve goals of individual care plans and patient involvement. Furthermore, it is crucial to increase awareness of the system approach to effects on care practice and procedures. This is relevant for future healthcare educational programs that could emphasize the importance of professional identity and communication to promote person-centered care.

### Strengths and limitations

To the best of our knowledge, no other Danish study has in recent times followed older patients and assessed their clinical care trajectory in a period before admission, during the hospital stay and after discharge.

This study focuses on healthcare professionals’ practice. A strength of this study is the inclusion of various professions from different sectors of the healthcare system. However, conducting clarifying interviews with the involved healthcare professionals in the observation period was not always possible due to work flow and task prioritization.

Making use of an internal gatekeeper with a prominent role in the department may result ina risk of coercion due to power imbalance. However, the gate-keepers role was kept to a minimum and any trajectory, where the gate-keeper was actively involved professionally was omitted.

Screening the patient flow management screen before approaching the patient may raise an ethical dilemma. However, practicalities made it impossible to gain access and otherwise recruit. Furthermore, the study obtained organizational acceptance and formal improvement to be conducted.

For ethical reasons, the most vulnerable older adults, such as those who have dementia, were excluded. However, if the more vulnerable patients had been included, the results may have demonstrated an even more limited patient involvement and distortion of healthcare professional and patient relationships.

Member-checking of the preliminary results with e.g. the HCps observed, would have benefitted the study further. However, the authors thoroughly made use of triangulation in the analysis process as described.

Applying Habermas as a theoretical framework in this discussion highlights the importance of communication and dialogue. However, Habermas’s critics emphasize that his theories do not sufficiently include culture, individual morals and values [48], which also affect the care practice. We suggest that more research is needed to investigate the clinical care trajectory for older adults including other aspects such as working cultures in context.

## Conclusion

Healthcare professionals’ interactions and practice are increasingly dominated by technical knowledge and market rationality as a result of NPM and numerous organizational changes in the healthcare system. The development of new professionalism with new tasks and a more specialised, evidence-based practice changes the relationship between healthcare professionals and patients, resulting in a clash between the system and healthcare practice intended to deliver person-centered care acknowledging the lifeworld of patients and relatives. However, there are more factors than merely system changes and societal developments contributing to healthcare professionals’ practice. While the system contributes to procedures and uniformity, healthcare professionals’ lifeworld enables every healthcare professional to interact individually and flexibly with patients within the given framework of organizational structures and resources. The lifeworld of professionals contributes to the nuanced and complex practice within clinical care trajectories. This study illustrates the need for better communicative practice based on equalized power to accommodate power distortion between healthcare professionals and patients. Such practices and procedures should be embedded immediately in educational programs to ensure a more appropriate and less paternalistic approach to healthcare. Furthermore, organizational structures and decision-makers should allocate resources to prioritize dialogue in daily interations to ensure better quality of the healthcare practice. This prioritization would help avoid overlooking patients’ needs and power clashes between professions.

## Data Availability

The datasets generated and analysed during the current study are not publicly available due to individual privacy but are available from the corresponding author on reasonable request.

## Abbreviations

ED: Emergency department
GP: General practitioner
NPM: New public management
SRQR: Standards for reporting qualitative research
WHO: World health organisation

## Footnotes

1 Which type of healthcare professionals are observed is not given on before-hand but guided by the clinical care trajectory. Thus, the helhcare professionals potentially recruited are those involved in the older person’s clinical care trajectory.

2 All new trajectories were initiated within the acute medical ward of the ED.
